# Initial therapeutic strategy of invasive candidiasis for intensive care unit patients: a retrospective analysis from the China-SCAN study

**DOI:** 10.1186/s12879-017-2207-1

**Published:** 2017-01-23

**Authors:** Na Cui, Hao Wang, Longxiang Su, Haibo Qiu, Ruoyu Li, Dawei Liu, on behalf of the China-SCAN Team

**Affiliations:** 1 Department of Critical Care Medicine, Peking Union Medical College Hospital, Peking Union Medical College & Chinese Academy of Medical Sciences, Beijing, 100730 China; 2 Nanjing Zhongda Hospital, Southeast University School of Medicine, Nanjing, China; 3 Peking University First Hospital, Research Center for Medical Mycology, Peking University, Beijing, China

**Keywords:** Invasive Candida infection, Initial therapeutic strategy, Clinical outcome, ICU

## Abstract

**Background:**

To investigate the impact of initial antifungal therapeutic strategies on the prognosis of invasive *Candida* infections (ICIs) in intensive care units (ICUs) in China.

**Methods:**

A total of 306 patients with proven ICIs in the China-SCAN study were analyzed retrospectively. Empiric, pre-emptive, and targeted therapy were adopted based on starting criteria including clinical, microbiological, and other conventional prediction rules. The primary outcome was hospital mortality and the secondary endpoints were duration days in ICU and duration days in hospital. The global responses (clinical and microbiological) at the end of the empirical therapy were also assessed.

**Results:**

A total of 268/306 (87.6%) ICI patients received antifungal therapy, including 142/268 (53.0%) initial empirical therapy, 53/268 (19.8%) initial pre-emptive therapy, and 73/268 (27.2%) initial targeted therapy. Compared with the initial empirical antifungal therapy and targeted antifungal therapy, patients with initial pre-emptive antifungal therapy had significantly less clinical remission [11/53 (21.2%) *vs*. 61/142 (43.3%) *vs*. 22/73 (30.1%), *P* = 0.009], higher ICU [26/53 (57.8%) *vs*. 42/142 (32.2%) *vs*. 27/73 (43.5%), *P* = 0.008] and hospital mortality [27/53 (60.0%) *vs*. 43/142 (32.8%) *vs*. 29/73 (46.8%), *P* = 0.004] and more microbiological persistence [9/53 (17.0%) *vs*. 6/142 (4.2%) *vs*. 9/73 (12.3%), *P* = 0.011]. Kaplan-Meier survival analysis revealed that ICI patients with initial pre-emptive antifungal therapy and targeted antifungal therapy were associated with reduced hospital duration compared with patients with initial empirical antifungal therapy after confirmation of fungal infection (log-rank test: *P* = 0.021). Multivariate regression analysis provided evidence that initial empirical antifungal therapy was an independent predictor for DECREASING the hospital mortality in ICI patients on ICU admission and at ICI diagnosis (odds ratio 0.327, 95% confidence interval 0.160–0.667, *P* = 0.002; odds ratio 0.351, 95% confidence interval 0.168–0.735, *P* = 0.006).

**Conclusions:**

The initial therapeutic strategy for invasive candidiasis was independently associated with hospital mortality. Prompt empirical antifungal therapy could be critical to decrease early hospital mortality.

**Trial registration:**

Clinicaltrials.gov NCT01253954 (retrospectively registration date: December 3, 2010)

## Background

Invasive fungal infection (IFI) is one of the most common in opportunistic infections for critically ill patients [1]. Recently, IFI incidences in intensive care units (ICUs) have increased year by year with improvements in hygiene, disease management, and life support technology [2]. Invasive candidiasis infection (ICI) is the most common and important part of IFI [3, 4]. Candidemia is responsible for 7–10% of nosocomial bloodstream infections, and *Candida* is located in the top five most common pathogens [5–7]. The 6.9 per 1000 candidemia incidence of ICU patients and 7.5% of prevalence of antifungal agents administered have been reported [8, 9]. ICI, mostly candidemia, increases the mortality rate, represents longer lengths of ICU or hospital stay, and uses a great deal of healthcare expenses and resources [3, 4, 10]. Thus, finding an appropriate treatment strategy for the best ICI treatment is an issue that needs to be resolved.

Anti-infection strategies for ICI are complex processes that include both clinical diagnosis and treatment; delayed treatments worsen the prognosis, drug abuse leads to antimicrobial drug selection pressure, and wasting resources is an important clinical problem. Faced with ICIs in critically ill patients, we urgently need to know whether giving early empirical antifungal therapy, then adjusting according to clinical diagnosis circumstances, or waiting for the microbiological evidence to determine the start of the antifungal treatment is acceptable or not. Additionally, knowing the types of evidence to collect before we begin antifungal therapy would benefit patients’ prognoses [direct culture detection or surrogate markers, such as *Candida* mannan antigens and anti-mannan antibodies and β-D-glucan, or polymerase-chainreaction (PCR) assays]. Although many experts and proposed guidelines suggest prophylaxis, empirical, pre-emptive, targeted, and other various initial antifungal treatment strategies, there is no currently available evidence for the effectiveness of previously mentioned treatments in ICU patients. The China Survey of Candidiasis in the ICU (China-SCAN) study was performed to assess the current incidence, mortality, pathogen spectrum, management, treatment, and risk factors for ICI in China’s ICUs [11]. The data from the China-SCAN study were used to investigate the impact of an initial antifungal therapeutic strategy on the prognosis of patients with ICIs in Chinese ICUs. We expect this study to provide a reasonable basis for how to select antifungal therapy strategies clinically.

## Methods

### Study design and patients involved

**Fig. 1:**
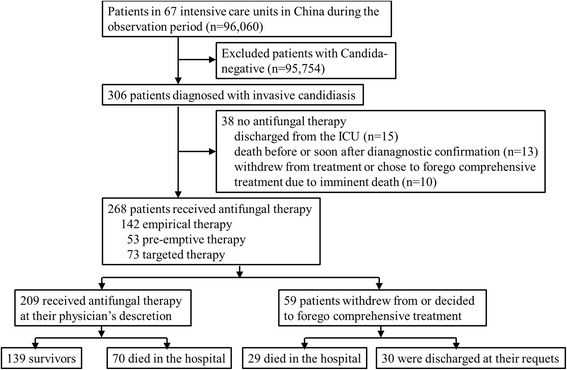
China-SCAN study patients distribution and flowchart for this study

The China-SCAN study is the largest observational study on prevalence of ICI in China and was conducted between November 2009 and April 2011 in 67 participating ICUs distributed throughout China. The details of this study, including the study population, inclusion, and exclusion criteria were previously published [11]. All the participating hospitals accepted the central ethics committee (Ethics Committee of Zhongda Hospital of Southeast University) review or conducted a further, independent, ethics review according to their own institutional policy. This was registered with ClinicalTrials.gov (NCT01253954). During the China-SCAN trial, 306 out of 96,060 ICU patients from 67 centers throughout China were diagnosed with ICIs by direct detection of fungal infection. A total of 268/306 (87.6%) patients finally received antifungal therapy. The remaining 59 patients withdrew from or decided to forego comprehensive treatment, 29 of whom died in the hospital and 30 of whom were discharged at their request. Based on the initial antifungal therapeutic strategies received, all the patients included were divided into empirical (*n* = 142), pre-emptive (*n* = 53), and targeted (*n* = 73) therapy groups. Based on the clinical prognosis of patients who died in the hospital or completed treatment and were discharged at their physician’s discretion, the population was further divided into survivor (*n* = 139) and non-survivor (*n* = 99) groups. Figure 1 presents a flowchart of patient screening and selection for this study.

### Evidence for antifungal treatment and initial antifungal therapy strategy

#### Definition of infection

Based on the guidelines for the diagnosis and management of candidiasis issued by the Infectious Diseases Society of America [12] and the European Society of Clinical Microbiology and Infectious Diseases [13], the evidence used to treat antifungal infection in the China-SCAN trial was divided into the following four categories:

- Clinical signs of infection diagnosed by the opinion of treating physician had to meet two or more of the following clinical features [14]: (i) body temperature ≥ 38 or < 36 °C; (ii) respiratory rate ≥ 30 breaths per min; (iii) pulse rate ≥ 120 beats per min; and (iv) abnormal total peripheral white blood cell counts ≥ 10,000 or < 4000 per mm^3^, or immature neutrophils > 15%.
- Risk factors of fungal infection: etiology-related factors such as neutropenia, hematological diseases, transplantation, surgery, and tumors; treatment-related factors, such as parenteral nutrition, broad-spectrum antibiotics, venous indwelling catheters, and other factors causing increased rate of fungal infection.
- Microbiological evidence: Indirect evidence of fungal infections: serological parameters including Galactomannan and β-D-glucan. The fungal colonization index was calculated [15]. The *Candida* colonization index (CCI) was calculated as the ratio of the number of culture-positive surveillance sites to the total number of sites cultured. A CCI ≥ 0.5 was considered as indirect evidence of invasive candidiasis in this study. Direct detection of fungal infections: blood and tissue specimens obtained from normally sterile sites were cultured. One thing to be mentioned, fungal combined bacterial infection, or even viral infection occurred in one patient maybe exist. So co-infection were not be systematically excluded.

#### Treatment options and outcomes

By combining evidence for the antifungal treatments mentioned above and actual clinical situations in mainland China hospitals, initial antifungal therapy began when patients met the following clinical features based on personal choice of the physician: (1) Empirical antifungal therapy: the patients had clinical signs of infection with any of the risk factors of fungal infection; (2) Pre-emptive antifungal therapy: the patients must have at least one indirect evidence of a fungal infection; (3)Targeted antifungal therapy: in addition to meeting the conditions for empirical antifungal therapy, the patients must have one direct evidence of a fungal infection.

Complete clinical efficacy was defined as elimination of all signs and symptoms of ICI, and accompanying radiographic resolution; partial efficacy was defined as improvement rather than complete elimination. Microbiological elimination was defined as negative culture from the original infection site.

### Statistical analysis

Quantitative data with normal distributions are denoted as the means ± standard deviations. Student’s *t* test or one-way analysis of variance (ANOVA) was performed to compare the means between the different groups. Abnormally distributed quantitative data were denoted using medians (interquartile ranges) and the rank-sum test was used to analyze these values. Data of unordered categories were denoted by rate, and differences in the rate between two groups were examined through a chi-square test. Survival curves for ICU and hospital duration were estimated using the Kaplan-Meier method, and a log rank (Mantel-Cox) test was used to estimate differences among these three antifungal therapies. Correlation between variables was tested by Spearman’s coefficient of rank correlation. Variables found to be significantly associated with mortality with a *P*-value < 0.01 at univariate analysis were introduced into a logistic stepwise regression model. All significant variables with collinearity were excluded from the regression model. When deciding which covariates to retain as candidate predictors for the multivariable model, we considered the clinical relevance of each covariate. The adjusted odds ratio of dying and the 95% confidence interval for all independent factors associated with mortality were calculated. Statistical analyses were performed with SPSS 16.0 (SPSS, Chicago, IL, USA), and a *P* value of < 0.05 was considered significant.

## Results

### Baseline characteristics and risk factors of fungal infection in ICI patients receiving antifungal therapy

Table 1 presents the baseline characteristics of the patients whose data were analyzed in this study. As shown in the table, there were no significant cross-group differences in terms of gender, body weight, hospital stays before ICU admission, ICU and hospital stays before candidiasis onset, Acute Physiology and Chronic Health Evaluation (APACHE) II score and sequential organ failure assessment (SOFA) score on ICU admission, and accompanying underlying diseases. However, the age of patents receiving initial targeted antifungal therapy was higher than other two groups. Table 2 lists the risk factors of fungal infection present within 2 weeks (3 months for immunosuppressants) prior to study entry, or at diagnosis, among 268 patients with ICI who received antifungal therapy in the China-SCAN study. The indwelling arterial catheterized in a significantly lower proportion of patients in targeted therapy group compared with other two groups (*P* < 0.05). The immunocompromised state, antibiotic therapy, clinical procedures including life-sustaining treatments ≥ 24 h, central venous catheterization, drainage and urethral catheterization, gastrointestinal dysfunction, parenteral nutrition, and surgery showed no significant differences among the different therapy strategies.

**Table 1:** Baseline characteristics of 268 ICI patients receiving antifungal therapy in the China-SCAN study

Variables	Empirical *N* = 142	Pre-emptive *N* = 53	Targeted *N* = 73	*P*-value
Age, years	58.3 ± 21.6	58.3 ± 18.4	68.7 ± 16.2	0.001
Gender, *n* (%)				0.414
Male	98 (69.0)	40 (75.5)	47 (64.4)	
Female	44 (31.0)	13 (24.5)	26 (35.6)	
Body weight, kg	61.8 ± 10.7	63.5 ± 10.4	63.8 ± 10.1	0.473
Hospital stays before ICU admission, days	0.5 (10.0)	1.0 (4.5)	0.0 (11.5)	0.829
ICU stays before candidiasis onset, days	9.0 (17.0)	10.0 (14.5)	11.0 (33.0)	0.717
Hospital stays before candidiasis onset, days	16.5 (23.3)	12.0 (24.5)	16.0 (41.0)	0.461
Underlying disease, *n* (%)				
Diabetic mellitus	28 (19.7)	13 (24.5)	21 (28.8)	0.318
Chronic cardiac dysfunction^a^	23 (16.2)	14 (26.4)	17 (23.3)	0.210
Chronic obstructive pulmonary disease	15 (10.6)	6 (11.3)	12 (16.4)	0.449
Chronic renal insufficiency^b^	12 (8.5)	5 (9.4)	13 (17.8)	0.108
Chronic hepatic insufficiency^c^	4 (2.8)	1 (1.9)	6 (8.2)	0.111
Solid tumor	26 (18.3)	7 (13.2)	17 (23.3)	0.353
Haematological malignancy	2 (1.4)	1 (1.9)	-	0.545
Illness severity at ICU admission				
APACHE II score	20.0 ± 9.2	20.9 ± 8.1	21.5 ± 8.6	0.517
SOFA score	7.2 ± 3.7	7.7 ± 3.4	6.8 ± 3.8	0.434

Continuous variables are expressed as the means ± SD or medians (IQR). All the other data are raw numbers (%)

*APACHE II* Acute Physiology and Chronic Health Evaluation II, *SOFA* sequential organ failure assessment

^a^All patients corresponding to the New York Heart Association (NYHA) standards of level II or higher

^b^All patients receiving long-term hemodialysis

^c^As described according to APACHE II criteria: biopsy-proven cirrhosis and documented portal hypertension; episodes of past upper gastrointestinal bleeding attributed to portal hypertension; or prior episodes of hepatic failure/encephalopathy/coma

**Table 2:** Risk factors of fungal infection present within 2 weeks (3 months for immunosuppressants) prior to study entry, or at diagnosis, among 268 ICI patients receiving antifungal therapy in the China-SCAN study

Variables	Empirical *N* = 142	Pre-emptive *N* = 53	Targeted *N* = 73	*P*-value
Immune compromised, *n* (%)				
Immunosuppressant therapy^a^	5 (3.5)	3 (5.7)	5 (6.8)	0.535
HIV infection	1 (0.7)	1 (1.9)	-	0.476
Neutropenia	3 (2.1)	-	-	0.260
Previous antibiotic therapy, *n* (%)^b^				
cephalosporins	38 (26.8)	9 (17.0)	12 (16.4)	0.138
carbapenems	49 (34.5)	18 (34.0)	20 (27.4)	0.554
pennicillins	62 (43.7)	21 (39.6)	30 (41.1)	0.858
quinolones	17 (12.0)	8 (15.1)	9 (12.3)	0.839
glycopeptides	26 (18.3)	10 (18.9)	12 (16.4)	0.925
Life-sustaining treatments ≥ 24 h				
Invasive mechanical ventilation	113 (79.6)	47 (88.7)	60 (82.2)	0.337
vasopressor	47 (33.1)	12 (22.6)	19 (26.0)	0.286
Renal replacement therapy	8 (5.6)	5 (9.4)	8 (11.0)	0.345
Catheterization, *n* (%)^c^				
central venous	113 (79.6)	45 (84.9)	66 (90.4)	0.122
indwelling arterial	27 (19.6)	13 (25.0)	6 (8.5)	0.040
drainage tube	52 (36.6)	26 (49.1)	23 (31.5)	0.124
urethral	104 (73.8)	43 (81.1)	53 (73.6)	0.534
Gastrointestinal dysfunction, *n* (%)^d^	72 (50.7)	32 (60.4)	45 (61.6)	0.229
Total parenteral nutrition, *n* (%)	70 (49.3)	30 (56.6)	41 (56.2)	0.513
Surgery, *n* (%)	59 (41.5)	24 (45.3)	25 (34.7)	0.457
Abdominal	33 (23.2)	20 (37.7)	18 (24.7)	0.114

Continuous variables are expressed as the means ± SD or medians (IQR). All the other data are raw numbers (%)

^a^Variables included steroid therapy, which defined as > 0.5 mg/(kg · day)^−1^ prednisone over 1 month (*n* = 7), cancer chemotherapy (*n* = 6), post-solid organ transplant immunosuppression (*n* = 1), allogeneic bone marrow transplantation or allogeneic haematopoietic stem cell transplantation (*n* = 1), and tumor necrosis factor therapy (*n* = 1) within 3 months prior to study entry

^b^All patients receiving systemic drug therapy for ≥ 3 days within 2 weeks prior to study entry

^c^Variables included patients who required treatments were catheterized within 2 weeks of the first positive sample no matter the catheter was removed or not before diagnosis

^d^Variables included hemorrhage, food intolerance, perforation, surgery, acalculouscholecystitis or intra-abdominal hypertension

### Microbiological and clinical characteristics of ICI patients receiving antifungal therapy

The candidiasis source of this study population was mainly bloodstream infections, which accounted for 95.9% (257/268) of infections. Comparing the sources of candidiasis among the three therapy strategies, only the targeted group had more abdominal candidiasis infections (*P* = 0.026). *C. albicans* (107, 39.9%), *C. parapsilosis* (42, 15.7%), *C. tropicalis* (37, 13.8%), and *C. glabrata* (37, 13.8%) were the most prevalent species isolated, with mixed infection were identified in 2/107 (1.9%), 1/42 (2.4%), 1/37 (2.7%), and 1/37 (2.7%) patients who received antifungal therapy in this study. Further *Candida* species were identified in < 2% of samples. 22 (8.2%) fungal isolates with proven yeast forms that were most suggestive of *Candida* spp. could not be further characterized. Based on microbiological determination of MIC, the susceptibility to antifungal treatment was 89.9% (241/268) and the complete resistance rate was 6% (15/268). There were no significant differences in the pathogenic *Candida* species, susceptibility and duration of initial antifungal medication between the three different treatment groups. However, the initial antifungal agents differed between the three groups, more caspofungin (37.7%) were used in pre-emptive group while more voriconazole (28.8%) were used in targeted group. Compared with empirical and targeted therapy group, the pre-emptive group showed significantly higher microbiological persistence (*P* = 0.011) and adjustment probability after initial antifungal therapy (*P* < 0.05).

In addition of microbiological characteristics, the clinical procedures and treatment outcomes of the 268 ICI patients who received antifungal therapy were also listed in the Table 3. There were no significant differences in the APACHE II score, vasopressor administration, and catheterization at ICI diagnosis between the three different strategy groups. The SOFA score at ICI diagnosis and immunopotentiation therapy including immunoglobulin and thymosin were significantly higher while the central venous catheter removed within 48 h after first positive sample obtained were significantly lower in pre-emptive group than in other two groups (*P* < 0.05).

**Table 3:** Microbiological and clinical characteristics among 268 ICI patients receiving antifungal therapy in the China-SCAN study

Variables	Empirical *N* = 142	Pre-emptive *N* = 53	Targeted *N* = 73	*P*-value
Source of candidiasis, *n* (%)^a^				
Blood culture	138 (97.2)	51 (96.2)	68 (93.2)	0.366
Proven catheter-related^b^	12 (8.5)	10 (18.9)	7 (6.8)	0.056
Abdominal	2 (1.4)	1 (1.9)	6 (8.2)	0.026
Intracranial	1 (0.7)	1 (1.9)	1 (1.4)	0.762
Pulmonary	-	1 (1.9)	-	0.131
Pleural	1 (0.7)	-	-	0.641
Pathogenic *Candida* species, n (%)^c^				
*C. albicans*	56 (39.4)	20 (37.7)	31 (42.5)	0.854
*C.tropicalis*	18 (12.7)	10 (18.9)	9 (12.3)	0.490
*C.glabrata*	18 (12.7)	11 (20.8)	8 (11.0)	0.247
*C.parapsilosis*	21 (14.8)	7 (13.2)	14 (19.2)	0.605
Uncategorized	11 (7.7)	5 (9.4)	6 (8.2)	0.930
Initial antifungal therapy^d^				
Categories, *n* (%)^e^				
fluconazole	61 (43.0)	16 (30.2)	24 (32.9)	0.160
caspofungin	27 (19.0)	20 (37.7)	17 (23.3)	0.024
voriconazole	20 (14.1)	8 (15.1)	21 (28.8)	0.025
Susceptibility, *n* (%)				
susceptible	125 (88.0)	48 (90.6)	68 (93.2)	0.490
completely resistant	9 (6.3)	3 (5.7)	3 (4.1)	0.797
Duration, days	8.5 (10.0)	6.0 (9.8)	7.0 (12.0)	0.932
Drug Adjustment, *n* (%)	67 (47.2)	35 (66.0)	30 (41.1)	0.017
Procedures at diagnosis, n (%)				
APACHE II score	19.5 ± 8.1	20.9 ± 8.8	21.2 ± 7.4	0.517
SOFA score	6.7 ± 3.5	8.2 ± 4.1	6.2 ± 3.7	0.010
Vasopressor	44 (31.0)	18 (34.0)	21 (28.8)	0.824
Catheterization, *n* (%)^f^				
central venous	90 (64.7)	37 (69.8)	58 (79.5)	0.086
indwelling arterial	19 (13.8)	9 (17.3)	5 (7.0)	0.202
drainage tube	37 (26.8)	21 (42.0)	21 (29.6)	0.133
urethral	100 (70.9)	41 (77.4)	52 (72.2)	0.668
Central venous catheter removed within 48 h after first positive sample obtained, *n* (%)	102 (71.8)	28 (52.8)	49 (67.1)	0.043
Drainage catheter removed within 48 h after first positive sample obtained, *n* (%)	16 (11.3)	6 (11.3)	8 (11.0)	0.997
Immunopotentiation therapy, *n* (%)^g^	55 (38.7)	32 (60.4)	34 (46.6)	0.025
Microbiological evaluation, *n* (%)				
Eradication	73 (51.4)	27 (50.9)	36 (49.3)	0.958
Persistence	6 (4.2)	9 (17.0)	9 (12.3)	0.011
Clinical resolution, *n* (%)				0.045
Complete remission	61 (43.3)	11 (21.2)	22 (30.1)	0.009
Improvement	105 (74.5)	35 (67.3)	53 (72.6)	0.613
Clinical outcome				
ICU mortality, *n* (%)^h^	42 (32.1)	26 (57.8)	27 (43.5)	0.008
Hospital mortality, *n* (%)^h^	43 (32.8)	27 (60.0)	29 (46.8)	0.004
ICU duration, days	26.0 (30.5)	26.0 (26.0)	33.0 (44.8)	0.473
Hospital duration, days	44.0 (50.0)	32.0 (47.0)	44.0 (62.8)	0.357

Continuous variables are expressed as the means ± SD or medians (IQR). All the other data are raw numbers (%)

*APACHE II* Acute Physiology and Chronic Health Evaluation II, *SOFA* sequential organ failure assessment

^a^Diagnostic confirmation was based solely on at least one positive blood culture in 257 (95.9%) cases, on positive fluid culture from a normally sterile site (cerebral spinal fluid, ascitic fluid or pleural fluid) in 10 (3.7%) cases and on candidaemia combined with positive culture from a normally sterile site in 3 (1.1%) cases. Diagnosis was confirmed by histopathology in 1 patient (0.3%)

^b^The catheter-related blood stream infection was diagnosed according to a previous study (Mermel LA, Allon M, Bouza E, Craven DE, Flynn P, O’Grady NP, Raad II, Rijnders BJ, Sherertz RJ, Warren DK: Clinical practice guidelines for the diagnosis and management of intravascular catheter-related infection: 2009 Update by the Infectious Diseases Society of America. Clin Infect Dis 2009, 49: 1–45)

^c^ *C. albicans*,*C. tropicalis*,*C. glabrata*, and *C. parapsilosis* were the most prevalent species isolated. But some sample was not pure infected by single fungi from these four fungal. That is to say, mixed fungal infection existed. The proportion of *C. albicans*,*C. tropicalis*,*C. glabrata*, and *C. parapsilosis* combined others fungi were 1.9% (2/107), 2.7% (1/37), 2.7% (1/37), 2.4% (1/42), respectively. Uncategorized Candida species included the fungal isolates with proven yeast forms that were most suggestive of *Candida* spp. without further characterization (*n* = 22)

^d^First-line treatment comprised drug combination in 4/268 (1.5%) patients, 1 (0.9%) in empirical therapy group, 1 (1.2%) in pre-emptive therapy group, and 2 (2.6%) in targeted therapy group

^e^Fluconazole (101/268; 37.7%), caspofungin (64/268; 23.9%), and voriconazole (49/268; 18.3%) were the most widely used first-line agents

^f^All patients who were catheterized when the first positive samples were collected

^g^Variable included immunoglobulin and thymosin

^h^Among 268 ICI patients receiving anti-fungal therapy in China-SCAN study, 59 patients withdrew from or decided to forego comprehensive treatment, of whom 29 died in hospital and 30 were discharged at their requests. The ICU or hospital mortality rate listed here was based only on patients who died in hospital or completed treatment and were discharged at their physician’s discretion. The overall ICU mortality rate in empirical therapy, pre-emptive therapy, and targeted therapy groups decreased to 42 (29.6%), 26 (49.1%), and 27 (37.0%), respectively (*P* = 0.039); and the overall hospital mortality rate in these three groups decreased to 43 (30.3%), 27 (50.9%), and 29 (39.7%), respectively (*P* = 0.025) when all the 268 patients who received antifungal therapy were included

**Fig. 2:**
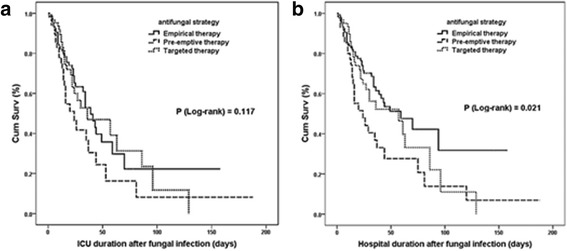
Kaplan-Meier analyses of survival probabilities after fungal infection among 268 ICI patients receiving antifungal therapy in the China-SCAN study. Survival was measured according to empirical, pre-emptive, and targeted therapy strategies. Survival time was censored on ICU discharge (**a**) or hospital discharge (**b**). Cum Surv: cumulative survivors

Among the 268 ICI patients who received antifungal therapy, the majority of patients (193/268, 72.0%) experienced clinical improvement, while complete clinical remission occurred in only (94/268, 35.1%) patients. In total, 59 patients withdrew from or decided to forego comprehensive treatment, of whom 29 died in hospital and 30 were discharged at their request. The overall ICU or hospital mortality rates lists here were based on the patients who died in the hospital or completed treatment and were discharged at their physician’s discretion. When all the 268 patients who received antifungal therapy were included, the overall ICU mortality rate in empirical, pre-emptive, and targeted therapy groups decreased to 42 (29.6%), 26 (49.1%), and 27 (37.0%), respectively (*P* = 0.039); and the overall hospital mortality rate in these three groups decreased to 43 (30.3%), 27 (50.9%), and 29 (39.7%), respectively (*P* = 0.025). Although duration of hospital stay and ICU stay after infection did not significantly differ among the three groups, the empirical therapy group had the highest clinical complete remission, lowest ICU mortality and hospital mortality compared to the other two groups (*P* < 0.01). From Kaplan-Meier analysis, the empirical therapy group had the highest survival probabilities after fungal infection for hospital duration (*P* = 0.021) rather than ICU duration (*P* = 0.117) (Fig. 2).

### Clinical differences between survivors and non-survivors receiving antifungal therapy

Analysis of the risk factors for mortality revealed a higher risk with increasing age and higher APACHE II score and SOFA score on ICU admission (*P* < 0.05). Among patients in the non-survivor group, the APACHE II score and SOFA score at the time of ICI diagnostic confirmation were significantly higher than those in the survivor group (*P* < 0.01). Additionally, vasopressor administration and central venous catheterization at ICI diagnosis occurred in a significantly higher proportion of patients in the non-survivor group compared with patients in the survivor group (*P* < 0.05). More importantly, the choice of initial antifungal therapy strategy differed significantly between the two groups (*P* = 0.004), more patients received empirical antifungal therapy in survivors than in non-survivors (*P* < 0.05) (Table 4).

**Table 4:** Risk factors for hospital mortality in 268 ICI patients receiving antifungal therapy in the China-SCAN study based only on patients who died in hospital or completed treatment and were discharged at their physician’s discretion

Variables	Survivors *N* = 139	Non-survivors *N* = 99	*P*-value
Age, years	58.6 ± 20.6	66.0 ± 19.8	0.006
Gender, *n* (%)			0.259
Male	91 (65.5)	72 (72.7)	
Female	48 (34.5)	27 (27.3)	
Body weight, kg	62.2 ± 10.4	62.4 ± 11.0	0.891
APACHE II score on ICU admission	19.0 ± 8.9	22.5 ± 8.4	0.002
SOFA score on ICU admission	6.6 ± 3.5	7.9 ± 3.8	0.008
Underlying disease, *n* (%)			
Diabetic mellitus	25 (18.0)	28 (28.3)	0.081
Chronic cardiac dysfunction^a^	26 (18.7)	26 (26.3)	0.203
Chronic obstructive pulmonary disease	16 (11.5)	15 (15.2)	0.439
Chronic renal insufficiency^b^	11 (7.9)	16 (16.2)	0.062
Chronic hepatic insufficiency^c^	4 (2.9)	7 (7.1)	0.208
Solid tumor	22 (15.8)	25 (25.3)	0.098
Haematological malignancy	1 (0.7)	2 (2.0)	0.572
Immune compromised, *n* (%)			
Immunosuppressant therapy^d^	4 (2.9)	6 (6.1)	0.327
HIV infection	-	1 (1.0)	0.416
Neutropenia	1 (0.7)	2 (2.0)	0.572
Source of candidiasis, *n* (%)^e^			
Blood culture	135 (97.1)	96 (97.0)	0.999
Other sterile site	5 (3.6)	5 (5.1)	0.745
Pathogenic *Candida* species, *n* (%)^f^			0.321
*C. albicans*	55 (39.6)	38 (38.4)	0.893
*C.tropicalis*	21 (15.1)	11 (11.1)	0.443
*C.glabrata*	15 (10.8)	18 (18.2)	0.128
*C.parapsilosis*	20 (14.4)	18 (18.2)	0.475
Initial antifungal therapy			
Strategies, *n* (%)			0.004
empirical therapy	66 (47.5)	35 (35.4)	0.041
pre-emptive therapy	37 (26.6)	35 (35.4)	0.155
Targeted therapy	36 (25.9)	29 (29.3)	0.658
Categories, *n* (%)^g^			0.348
fluconazole	54 (38.8)	34 (34.3)	0.499
caspofungin	34 (24.5)	21 (21.2)	0.640
voriconazole	22 (15.8)	25 (25.3)	0.098
Susceptibility, *n* (%)			
susceptible	122 (87.8)	89 (89.9)	0.682
completely resistant	9 (6.5)	6 (6.1)	0.999
Duration, days	8.0 (10.0)	6.0 (11.0)	0.428
Drug Adjustment, *n* (%)	62 (44.6)	54 (54.5)	0.149
Procedures within 2 weeks prior to diagnosis, *n* (%)			
Life-sustaining treatments ≥ 24 h			
Invasive mechanical ventilation	109 (78.4)	87 (87.9)	0.084
vasopressor	38 (27.3)	33 (33.3)	0.389
Renal replacement therapy	7 (5.0)	12 (12.1)	0.055
Catheterization, *n* (%)^h^			
central venous	113 (81.3)	87 (87.9)	0.210
indwelling arterial	24 (17.8)	21 (21.9)	0.501
drainage tube	53 (38.1)	38 (38.4)	0.999
urethral	101 (73.2)	81 (81.8)	0.160
Gastrointestinal dysfunction, *n* (%)^i^	79 (56.8)	54 (54.5)	0.791
Total parenteral nutrition, *n* (%)	70 (50.4)	54 (54.5)	0.599
Surgery, n (%)	60 (43.2)	35 (35.7)	0.283
Abdominal	39 (28.1)	25 (25.3)	0.659
Procedures at diagnosis, *n* (%)			
APACHE II score	18.8 ± 8.5	22.7 ± 7.0	0.000
SOFA score	6.4 ± 3.6	7.8 ± 3.8	0.003
Vasopressor	37 (26.6)	39 (39.4)	0.048
Catheterization, *n* (%)^j^			
central venous	88 (64.2)	76 (77.6)	0.031
indwelling arterial	20 (14.8)	13 (13.5)	0.850
drainage tube	40 (29.6)	32 (34.0)	0.563
urethral	97 (70.3)	79 (79.8)	0.132
Central venous catheter removed within 48 h after first positive sample obtained, *n* (%)	92 (66.2)	70 (70.7)	0.484
Drainage catheter removed within 48 h after first positive sample obtained, n (%)	19 (13.7)	8 (8.1)	0.216
Immunopotentiation therapy, *n* (%)^k^	55 (39.6)	51 (51.5)	0.085

Continuous variables are expressed as the means ± SD or medians (IQR). All the other data are raw numbers (%)

*APACHE II* Acute Physiology and Chronic Health Evaluation II, *SOFA* sequential organ failure assessment

^a^All patients corresponding to the NYHA standards of level II or higher

^b^All patients receiving long-term hemodialysis

^c^As described according to the APACHE II criteria: biopsy-proven cirrhosis and documented portal hypertension; episodes of past upper gastrointestinal bleeding attributed to portal hypertension; or prior episodes of hepatic failure/encephalopathy/coma

^d^Variables included steroid therapy, which defined as >0.5 mg/(kg · day)^−1^ prednisone over 1 month (*n* = 7), cancer chemotherapy (*n* = 6), post-solid organ transplant immunosuppression (*n* = 1), allogeneic bone marrow transplantation or allogeneic haematopoietic stem cell transplantation (*n* = 1), and tumor necrosis factor therapy (*n* = 1) within 3 months prior to study entry

^e^Diagnostic confirmation of other sterile sites was based on positive fluid culture from a normally sterile site (cerebral spinal fluid, ascitic fluid or pleural fluid) in 10 (3.7%) cases and on histopathology in one patient (0.3%); Diagnosis was confirmed by candidaemia combined with positive culture from a normally sterile site in 3 (1.1%) cases

^f^ *C. tropicalis*, *C. glabrata*, *and C. parapsilosis* were the most prevalent non-*C. albicans* species isolated, which were identified in >98% of samples

^g^First-line treatment comprised a single agent in 264/268 (98.5%) patients, most commonly fluconazole (101/268;37.7%), caspofungin (64/268; 23.9%), and voriconazole (49/268; 18.3%)

^h^Variables included patients who required treatments were catheterized within 2 weeks of the first positive sample no matter the catheter was removed or not before diagnosis

^i^Variable included haemorrhage, food intolerance, perforation, surgery, acalculouscholecystitis or intra-abdominal hypertension

^j^All patients who were catheterized when the first positive samples were collected

^k^Variable included immunoglobulin and thymosin

### Risk factors for hospital mortality in ICI patients receiving different antifungal therapy strategies

Variables found to be significantly associated with mortality with a *P* value < 0.01 at univariate analysis were introduced into a logistic stepwise regression model (Table 5). All significant variables with collinearity were excluded from the regression model. The first multivariate analysis was performed by introducing APACHE II and SOFA score on ICU admission as independent factors (Table 6) and the second one was performed by introducing APACHE II and SOFA score at ICI diagnosis as independent factors (Table 7). This was motivated by the fact that both the collinearity between APACHE II on ICU admission and at ICI diagnosis (*r* ^2^ = 0.51, *P* < 0.001) and SOFA score on ICU admission and at ICI diagnosis (*r* ^2^ = 0.48, *P* < 0.001) were high.

**Table 5:** Variables included in univariate logistic regression and significant associated (*P* < 0.01) with hospital mortality in 268 ICI patients receiving antifungal therapy in the China-SCAN study based only on patients who died in hospital or completed treatment and were discharged at their physician’s discretion

Variables	Survivors *N* = 139	Non-survivors *N* = 99	*P*-value
Age, years	58.6 ± 20.6	66.0 ± 19.8	0.006
APACHE II score on ICU admission	19.0 ± 8.9	22.5 ± 8.4	0.002
SOFA score on ICU admission	6.6 ± 3.5	7.9 ± 3.8	0.008
APACHE II score at diagnosis	18.8 ± 8.5	22.7 ± 7.0	0.000
SOFA score at diagnosis	6.4 ± 3.6	7.8 ± 3.8	0.003
Initial antifungal therapy strategies (empirical : pre-emptive : targeted)	66 : 37 : 36	35 : 35 : 29	0.004

*APACHE II* Acute Physiology and Chronic Health Evaluation II, *SOFA* sequential organ failure assessment

Continuous variables are expressed as the means ± SD or medians (IQR). All the other data are raw numbers (%)

**Table 6:** Multivariate analysis for hospital mortality in 268 ICI patients receiving antifungal therapy in the China-SCAN study with APACHE II and SOFA score on ICU admission as independent factors

Variables	B	SE	Wald’s coefficient	OR	95% CI for OR	*P*-value
					Lower Upper	
APACHE II score on ICU admission	0.046	0.016	8.389	1.047	1.015 1.080	0.004
Initial antifungal therapy strategies (empirical : pre-emptive : targeted)			10.127			0.006
Empirical : Pre-emptive	−1.117	0.363	9.449	0.327	0.160 0.667	0.002

*APACHE II* Acute Physiology and Chronic Health Evaluation II

**Table 7:** Multivariate analysis for hospital mortality in 268 ICI patients received antifungal therapy in the China-SCAN study with APACHE II and SOFA score at ICI diagnosis as independent factors

Variables	B	SE	Wald’s coefficient	OR	95% CI for OR	*P*-value
					Lower Upper	
Initial antifungal therapy strategies (empirical : pre-emptive : targeted)			8.068			0.018
Empirical : Pre-emptive	−1.047	0.377	7.706	0.351	0.168 0.735	0.006

The results showed that the APACHE II score on ICU admission (odds ratio 1.047, *P* = 0.004), which suggested risk factor for poor prognosis. Compared with pre-emptive therapy strategy, the empirical therapy may be a protective factor for poor prognosis on ICU admission and at ICI diagnosis (odds ratio 0.327, 95% confidence interval 0.160–0.667, *P* = 0.002; odds ratio 0.351, 95% confidence interval 0.168–0.735, *P* = 0.006).

## Discussion

IFI is one of the common infections in the ICU with a high level of mortality rate. A large number studies of anti-bacterial infection treatment suggested that prompt early empirical strategies of antibiotic usage is an important factor affecting anti-infection treatment effects and prognosis [16, 17]. However, whether this therapy strategy can be used in antifungal therapy remains unclear. In addition, previous studies relevant to the strategy of antifungal infections were carried out in fungal high-risk populations, including patients with non-fungal infections and confirmed fungal infections. Identification of patients at risk of *Candida* infections will inevitably impact the results for the appropriate antifungal treatment time. We selected 306 patients diagnosed with ICI from the China-SCAN study, which is the only study to assess antifungal therapy among patients in ICU diagnosed with ICIs. It excluded the high-risk patients with non-fungal infections, which maybe more objective and valuable. Additionally, we are not targeted to specific populations for analysis, such as bone marrow transplantation, leukemia, or liver transplantation. Therefore, prophylaxis was not discussed here. This study revealed that the initial empirical antifungal therapy can reduce hospital mortality and have longer hospital duration for patients with severe ICIs compared with the pre-emptive therapy. The appropriate initial antifungal therapeutic strategy chosen was an independent predicting factor for the prognosis of severe ICIs. It seemed that initial empirical antifungal therapy could be critical to decrease hospital mortality.

It has been recognized that early presumptive antifungal therapy is associated with improved clinical outcomes and reduced mortality of ICIs [18–20]. We also found similar results that empirical antifungal therapy helps to improve clinical complete remission, reduce the incidence of persistent fungal infection rate, and lower the hospital mortality in patients with severe ICIs compared with pre-emptive therapy. As is well known, signs and symptoms of candidiasis are nonspecific, and microbiological and imaging techniques lack sensitivity and specificity. Thus, early diagnosis of invasive candidiasis remains a challenge. Several placebo-controlled studies revealed that pre-emptive therapy could not reduce the incidence of ICIs or survival free of invasive fungal infection [21, 22]. Knitsch, et al showed that pre-emptive therapy was non-effective in preventing ICIs in high-risk surgical ICU patients with intra-abdominal infections due to the drug being administered too late [23]. In contrast with empirical therapy, pre-emptive therapy must have indirect evidence for fungal infections that the widespread use of antifungal therapies must be balanced against the risk of toxicity, costs, and the emergence of resistance. In this study, the patients included had confirmed ICIs rather than suspected fungal infections or non-fungal infections. Therefore, the “diagnostic” advantage of pre-emptive therapy and targeted therapy did not exist. This may be an important reason for early empirical antifungal therapy that could significantly improve clinical outcomes in patients with severe ICIs.

It has been shown that the lack of experience in treatment leading to improper drug selection could be an important risk factor for poor prognosis [24]. The results of this study showed that during the initial antifungal treatment, the empirical therapy group selected more fluconazole; the pre-emptive therapy group selected more caspofungin; and the targeted therapy group selected more voriconazole, which were partly consistent with the recommended guidelines [12, 13]. In addition, there was no statistical significance in antifungal drug sensitivity and the duration of drug administration among these three strategies. This result suggested that depending on the combination of local epidemiological survey data and guidelines, pre-emptive and targeted antifungal therapy had no advantage than empirical therapeutic strategy in clinical efficacy and safety. Interestingly, we also found that the drug adjustments for pre-emptive therapy after the initial antifungal therapy was significantly higher compared with the empirical therapy and targeted therapy groups (*P* = 0.017) in this study. It further confirmed the credibility and feasibility of empirical antifungal therapy, which could be another important reason for empirical therapy that significantly improves the clinical prognosis of ICI patients. Conversely, delaying the start time of the antifungal infection treatment is undoubtedly detrimental.

It has been demonstrated that critically ill ICU patients have complicated conditions. In the initial treatment strategy of the antifungal treatment evaluation process, including the basic conditions of the patient (severity of illness, invasive operation, gastrointestinal function, recent surgical history, previously selected types of antibiotics and antifungal drugs and other factors) is important as these conditions are likely to have an impact on prognosis and assessment. To further enhance the objectivity and accuracy of the assessment, all these risk factors are compared using statistical analysis. Diagnostic confirmation was based solely on at least one positive blood culture in 257 (95.9%) cases. Only 27 (10.8%) cases finally proved incidence of a catheter-related blood stream infection, and the reason for the lower incidence has been reported by Hu, et al on behalf of China-SCAN [25]. There was no significant difference among the three different strategies for abdominal sources of candidiasis. Maybe the absence of adequate abdominal source control caused late abdominal fungal infections, which led to a more targeted antifungal therapy after direct etiological evidence was found rather than early administration. Regarding the pathogenic *Candida* species, there was no significant difference [26]. Similar to other studies [27], the proportion of *non*-*albicans Candida* infection (60.1%) was higher than that of *Candida albicans* (39.9%) in this study population. Fortunately, the vast majority of *Candida* detected was susceptible to antifungal drugs. Because all patients in this study were diagnosed with ICI during hospitalization in ICU and the limitation of ICI occurrence influenced survival time after infection, it was more useful to compare the length of ICU and total hospitalization time to reflect the actual effectiveness of antifungal infection treatment. Therefore, Kaplan-Meier survival analysis revealed that ICI patients with initial pre-emptive antifungal therapy and initial targeted antifungal therapy were associated with a reduced hospital duration compared with patients with initial empirical antifungal therapy after confirmation of fungal infection (log-rank test: *P* = 0.021). This indicator was strongly demonstrated that early appropriate empirical antifungal therapy, which helps prolong the hospital survival time of patients with severe ICIs, may lay a solid foundation for reduced hospital mortality.

Our study has several limitations. First, this study only included patients diagnosed with ICI, which may provide a more clear influence of different strategies on ICIs. However, we did not evaluate the role of different strategies for the patients with a suspected ICI but no ICI confirmation. Second, although the China-SCAN study was a multi-center, observational study, the results of this study were based on statistical analysis rather than a prospective study on treatment strategies. Therefore, another RCT is needed to further confirm the direct effect of different therapeutic strategies on bacteriological and clinical prognosis. Third, 59 patients withdrew from or decided to forego comprehensive treatment in this study, 29 of whom died in the hospital and 30 were discharged at their request. The ICU or hospital mortality rate listed here was based only on patients who died in the hospital or completed treatment and were discharged at their physician’s discretion. We do not exclude the fact that this might have impacted the results of our statistical analysis.

## Conclusions

In summary, the initial antifungal treatment strategy formulation is an independent risk factor for the prognosis of patients with an ICI. Selecting the appropriate timing of the initial antifungal treatment could help improve the clinical cure rate, reduce the incidence of persistent fungal infection, and lower hospital mortality in patients with severe ICIs. This finding is consistent with the treatment strategy of resistance to bacterial infections. However, this conclusion has yet to be confirmed by a prospective RCT study in the future.

## Abbreviations

APACHE II score: Acute Physiology and Chronic Health Evaluation II score
China-SCAN: China Survey of Candidiasis in the ICU
ICIs: Invasive Candida infections
ICUs: Intensive care units
IFI: Invasive fungal infection
PCR: Polymerase-chain reaction
RCT: Randomized control trial
SOFA score: Sequential organ failure assessment score
